# Overexpression of AKR1C3 significantly enhances human prostate cancer cells resistance to radiation

**DOI:** 10.18632/oncotarget.10347

**Published:** 2016-06-30

**Authors:** Shao-Qian Sun, Xiaobin Gu, Xian-Shu Gao, Yi Li, Hongliang Yu, Wei Xiong, Hao Yu, Wen Wang, Yingbo Li, Yingqi Teng, Demin Zhou

**Affiliations:** ^1^ Department of Radiation Oncology, Peking University First Hospital, Peking University, Beijing, China; ^2^ State Key Laboratory of Natural and Biomimetic Drugs, School of Pharmaceutical Sciences, Peking University, Beijing, China; ^3^ Department of Radiation Oncology, Jiangsu Cancer Hospital Affiliated with Nanjing Medical University, Nanjing, China; ^4^ Tangshan People's Hospital, Hebei, China; ^5^ Beijing Reciproca Pharmaceutical Co. Ltd., Beijing, China

**Keywords:** AKR1C3, radioresistance, ROS, prostaglandinF2α, MAPK

## Abstract

Aldo-keto reductase 1C3(AKR1C3) is an enzyme involved in prostaglandins metabolism. Studies suggest that AKR1C3 has a pivotal role in the radioresistance of esophageal cancer and non-small-cell lung cancer, yet the role of AKR1C3 in prostate cancer cells radiation resistance has not yet been clarified. In our study, we established a stable overexpressing AKR1C3 cell line (AKR1C3-over) derived from the prostate cell line DU145 and its control cell line (Control). We conducted colony formation assay to determine the role of AKR1C3 in radioresistance and we used its chemical inhibitor to detect whether it can restored the sensitivity of the acquired tumor cells. Flow cytometry assay was carried out to detect IR-induced ROS accumulation. Elisa was adopted to dedect the concentration of PGF2α in the suspension of the cells after 6GY radiation. Western blotting was used to dedect the MAPK and PPAR γ. The results demonstrated that overexpression of AKR1C3 in prostate cancer can result in radioresistance and suppression of AKR1C3 via its chemical inhibitor indocin restored the sensitivity of the acquired tumor cells. According to the flow cytometry assay, ROS was decreased by 80% in DU145-over cells. Also overexpression of AKR1C3 could result in the accumulation of prostaglandin F2α (PGF2α), which can not only promote prostate cancer cell 's proliferation but also could enhance prostate cancer cells resistance to radiation and activated the MAPK pathway and inhibited the expression of PPARγ. In conclusion, we found that overexpression of AKR1C3 significantly enhanced human prostate cancer cells resistance to radiation through activation of MAPK pathway.

## INTRODUCTION

Prostate cancer (PCa) is a significant medical burden in developed countries and accounts for estimated 28,170 deaths in the United States and 94,000 deaths in Europe in 2012 [1]. Radiation therapy (RT) is one of the main treatments for localized PCa, however, radioresistance occurs in almost one third of PCa patients under curative dosage [2–4] Therefore, it is critical to investigate the genes and the mechanisms which cause radioresistance and to develop novel treatment approaches to overcome recurrence after RT in PCa patients.

Aldo-keto reductase family 1 member C3 (AKR1C3) is a hormones and prostaglandins enzyme which efficiently converts PGH2 to PGF2α and PGD2 to 9α, 11β-PGF2 [5–8] By converting PGD2 to 9α, 11β-PGF2, AKR1C3 prevents its spontaneous dehydrationand rearrangement forming the anti-proliferative and anti-inflammatory prostaglandin15d-PGJ2. Whereas 15d-PGJ2covalently reacts with a cysteineresidue in the ligand-binding domain of PPARγ, resulting in the activation of PPARγ [5, 9–13].

AKR1C3 is a radiation resistance gene in esophageal cancer [14] and non-smal-celll lung cancer [15]. Elevated expression of AKR1C3 has been reported to be associated with the progression and aggressiveness of PCa [16–18]. However, whether AKR1C3 participates in the radioresistance of PCa remains unclear. We hypothesized that overexpression of AKR1C3 in PCa could result in radioresistance.

In the current study, we established a stable overexpression cell line (AKR1C3-over) derived from the prostate cancer cell line DU145. Furthermore, we found that AKR1C3 overexpression could result in PCa DU145′s radioresistance,while the inhibition of AKR1C3 could restore the radiation sensitivity of the acquired tumor cells. We explored the mechanisms by detecting the amount of ROS. We also found that overexpression of AKR1C3 could lead to the accumulation of prostaglandin F2α which colud not only promote PCa cell 's proliferation but also enhance PCa cell's resistance to radition. Moreover, prostaglandin F2α could activate the MAPK pathway and inhibit the expression of PPARγ.

## RESULTS

### Establishment of stably transfected human prostate cancer cells and confirmation of AKR1C3 effect on the response of tumor cells to irradiation

**Figure 1:**
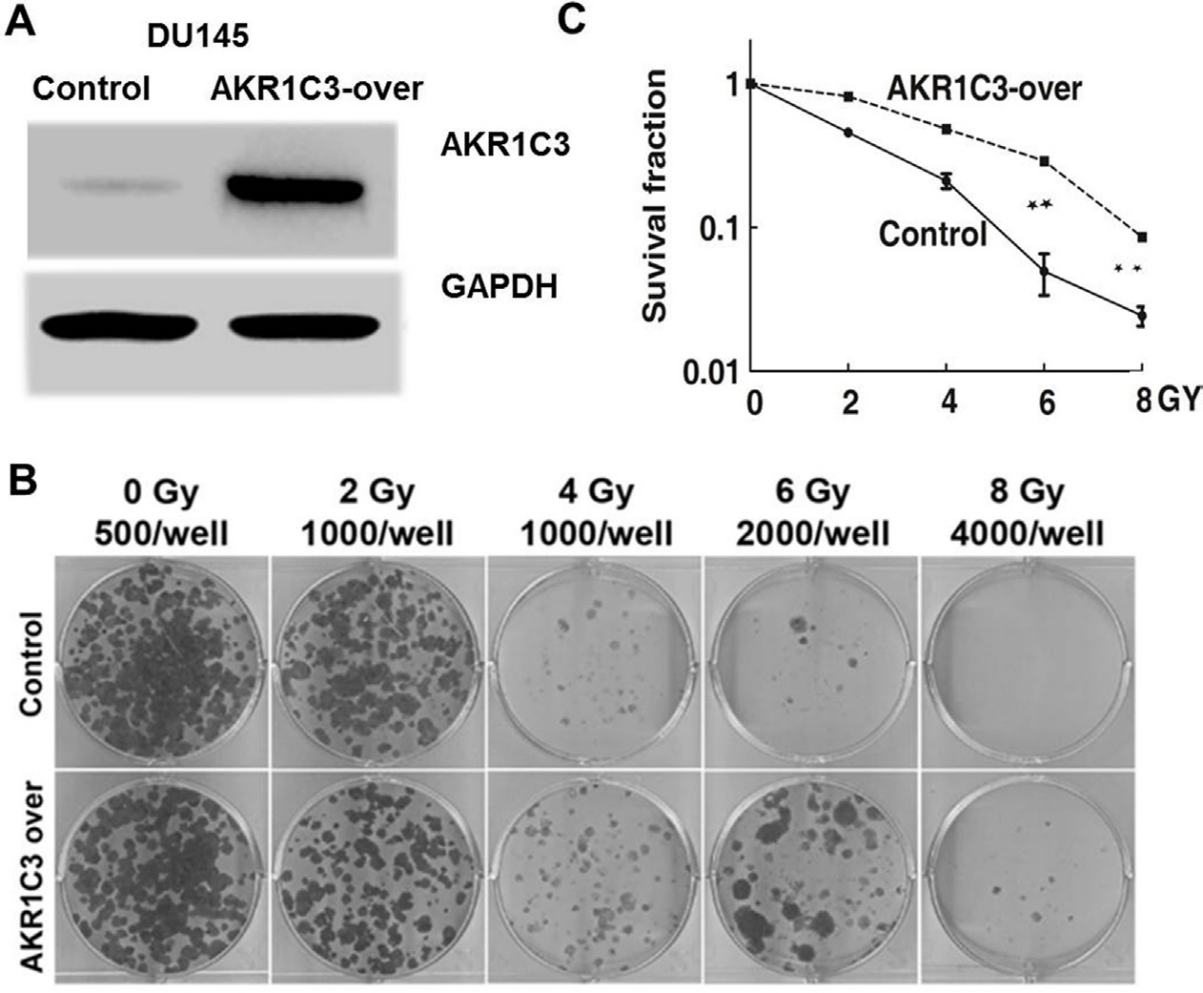
Cell-based evaluation of the role of AKR1C3 on the response of prostate cancer cells to irradiation (**A**) The constructions of stable overexpression of AKR1C3 in DU145 cells (AKR1C3-over) and the effects of AKR1C3 overexpression in DU145 cells were determined by Western blot. A scramble shRNA (CMV.) acted as a negative control (Control). (**B**) AKR1C3 overexpression in DU145 cells on the outcome of irradiation, dosed from 0 to 8 Gy was determined by colony-formation assay. The number of colonies formed in the wells increased when exposured at the same radiation doses (**C**) Dose-survival curve derived from the results of clonogenic assay.

The overepression of AKR1C3 in DU145 wereconstructed by BglII-CMV-BamHI (Control)and BamHI-AKR1C3-BamHI expression vectors (AKR1C3-over), cell culture, lentivector package, and stable transduction [19] and the effects of AKR1C3 overexpression on radioresistance were characterized. Western blot analysis was used to determine the levels of AKR1C3 protein expression in Control and AKR1C3-over stable transfectants. AKR1C3-over clone showed apparently elevated AKR1C3 protein expression (Figure 1A).

To test whether AKR1C3 was a radioresistace gene in PCa DU145 cells, we performed the colony formation assay. In the assay, it was found that AKR1C3-over cells allowed more colonies to survive than in the case of control cells upon the same dose of irradiation (*p* < 0.01) (Figure 1B and 1C). Clearly, it was the elevated expression of AKR1C3 that rendered AKR1C3-over cells substantially resistant to radiation. AKR1C3 confered resistance to radiation in PCa cells.

### Indomethacin, an inhibitor of AKR1C3 activity, overcomes radiation resistance

**Figure 2:**
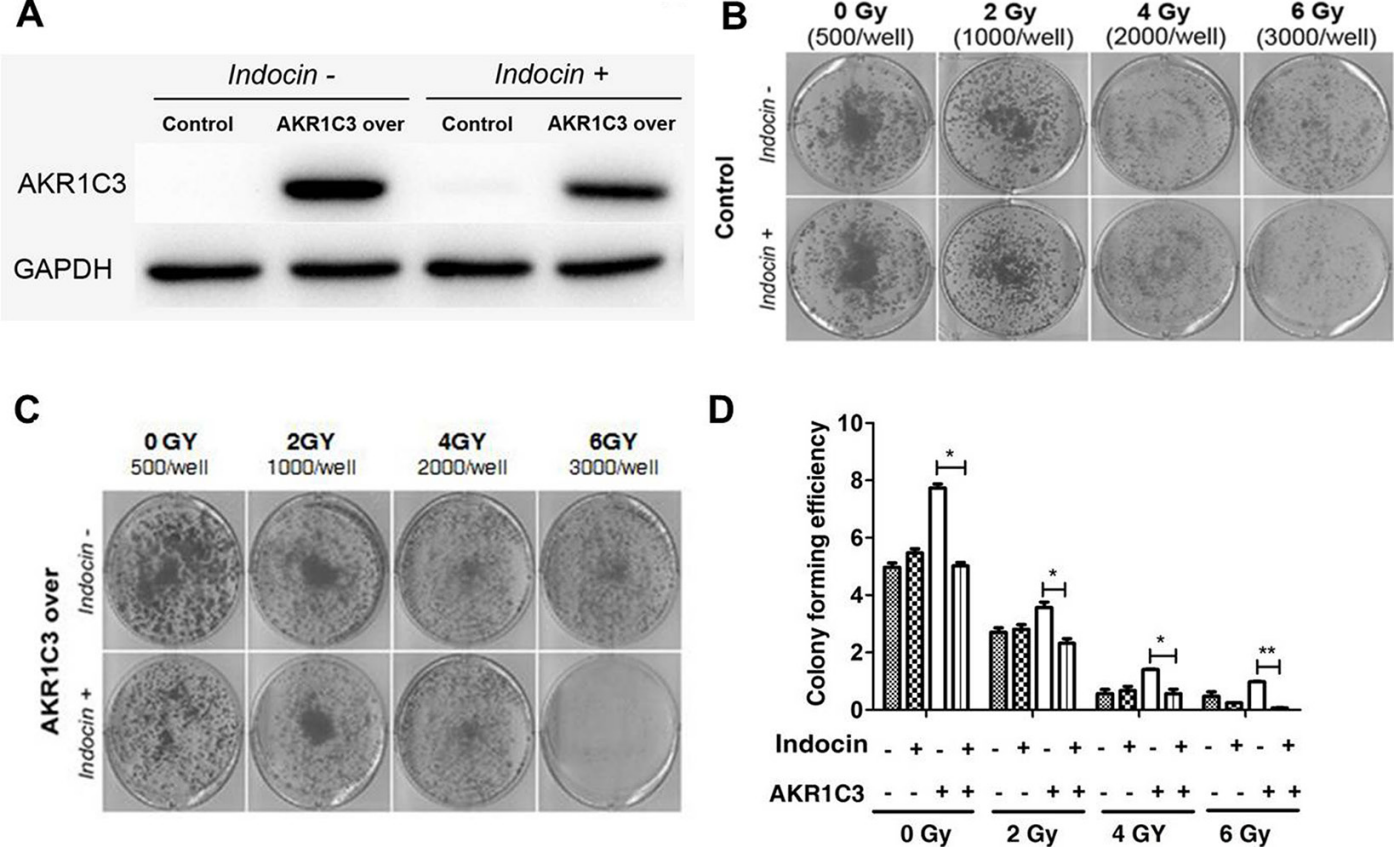
Indomethacin, an inhibitor of AKR1C3 activity, overcomes radiation resistance (**A**) AKR1C3-over cells and control cells were treated with or without 20 mmol/ indomethacin for 2 days, and western blotting was performed; (**B**, **C**) AKR1C3-over cells and Control cells were treated with or without 20 mmol/ indomethacin for 2 days, and clonogenic assay was performed; (**D**) Colony forming efficiency were calculated and results are presented as means SD of two experiments performed in duplicate.

Indomethacin, a NSAID, used for reducing fever, pain, and inflammation, has been shown to be able to inhibit AKR1C3 activity [20–22]. To further examine the role of AKR1C3 in radiation resistance, we used indomethacin to hinder AKR1C3 activation and examined the effects on the response of PCa cells to radiation treatment.As shown in Figure 2A, indomethacin has suppressed the expression of AKR1C3 protein. Combination of indomethacin with radiation significantly inhibited the growth of radiation-resistant cells (AKR1C3-over). The results were confirmed by clonogenic assay. As shown in Figure 2B and 2C, combination of indomethacin with radiation significantly inhibited the colony numbers in AKR1C3-over cells as well as the colony forming efficiency (Figure 2D). While in the control cells, there was no obvious effect.

### AKR1C3 canenhance DU145 cells resistance to t-BHP while indomethacin can overcome this effect

**Figure 3:**
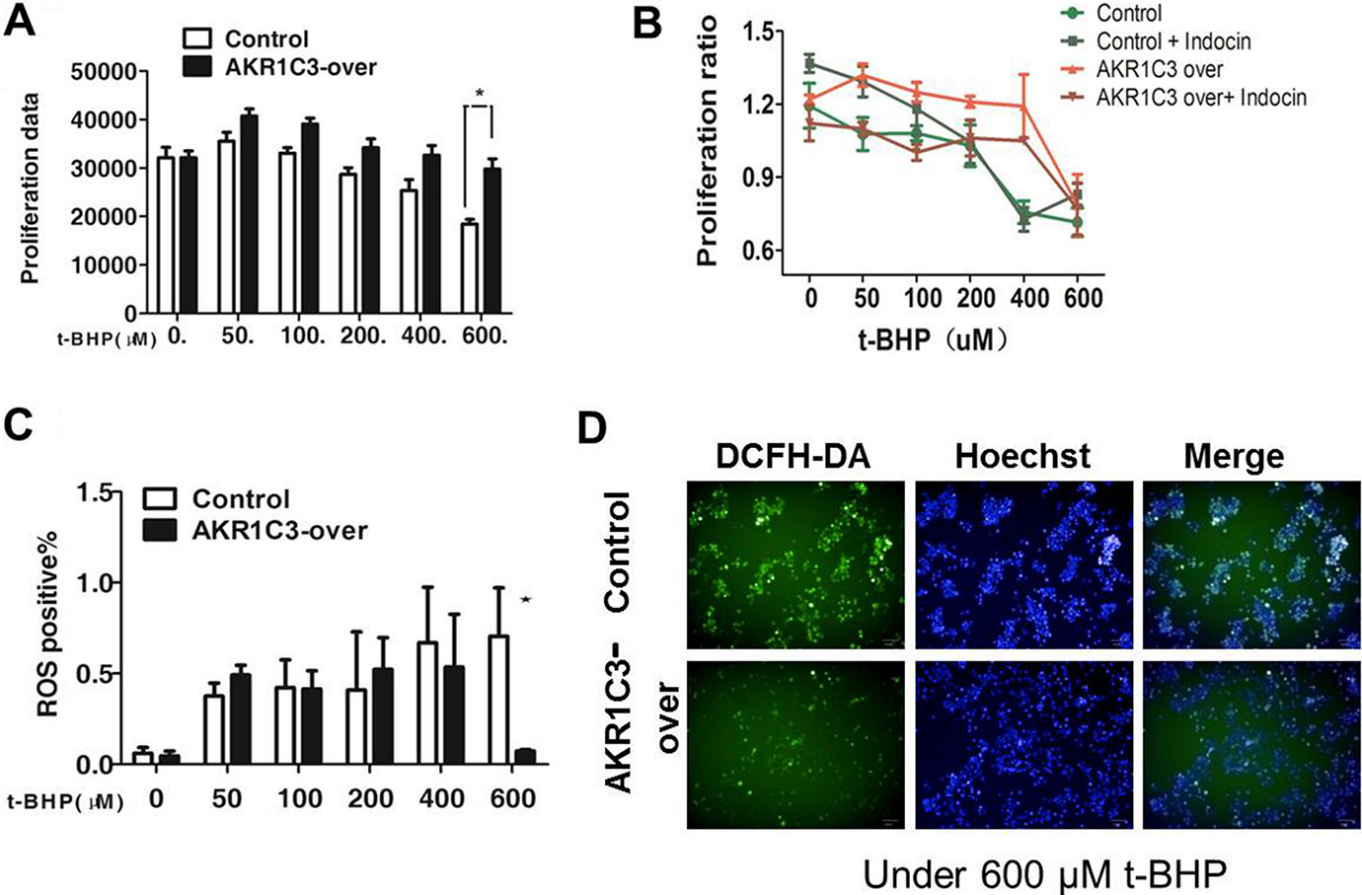
Mechanistic exploration of AKR1C3 as a cellular factor for protecting cells from irradiation damage (**A**) Control and AKR1C3-over cells were treated with a series concentration of t-BHP or DMSO for 1 h followed by cell proliferation assessment by CCK-8 assay. Data presented is the average of three experiments (SD), *P < 0.05, **P < 0.01. (**B**) AKR1C3-over cells,control cells, AKR1C3-over cells pretreated with 20 mmol/ indomethacin for 2 days and control cells pretreated with 20 mmol/ indomethacin for 2 days were treated with a series concentration of t-BHP or DMSO followed by cell proliferation assessment by CCK-8 assay. Data presented is the average of three experiments (SD), *P < 0.05, **P < 0.01. (**C**) Quantifications of the ROS levels in Control and AKR1C3-over cells prior to and 1 h after 600 μM t-BHP treatment. Data presented is the average of three experiments (SD), *P < 0.05, **P < 0.01. (**D**) Representative microscopic view of the accumulation of ROS in Control and AKR1C3-over cells stained by DFCH after 600 μM t-BHP treatment.

To explore the mechanism by which AKR1C3 mediated radioresistance, we compared the levels of cellular ROS between AKR1C3-over cells and control cells. We used tert-butyl hydroperoxide (t-BHP) to simulate radiation.We determined the proferation data in the presence of t-BHP and we found that under 600 μM t-BHP the proliferation data was almost 1.5-fold in AKR1C3-over than that in control cells (Figure 3A). However, combination of indomethacin with t-BHP significantly inhibited the growth of radiation -resistant AKR1C3-over cells. Collectively, these results suggested that inhibition of AKR1C3 by indomethacin reduced radiation-resistant tumor growth (Figure 3B), These results indicated that inhibition of AKR1C3 by indomethacin potentiated the cell killing effect of radiation.We used flow cytometry assay to dedect ROS. It was found that after 600 μM t-BHP treatment,there were approximately 5-fold less ROS in control cells than in AKR1C3-over cells (Figure 3C–3D). So AKR1C3 can enhance DU145 cells radioresistance to t-BHP while indomethacin can overcome this effect. Furthermore, AKR1C3 can alleviate the ROS in cells.

### PGF2α can not only promote prostate cancer cell's proliferation but also enhance prostate cancer cells resiatance to radition. The accumulation of PGF2α in AKR1C3-over cells activates the MAPK pathway and inhibits the expression of PPARγ

**Figure 4:**
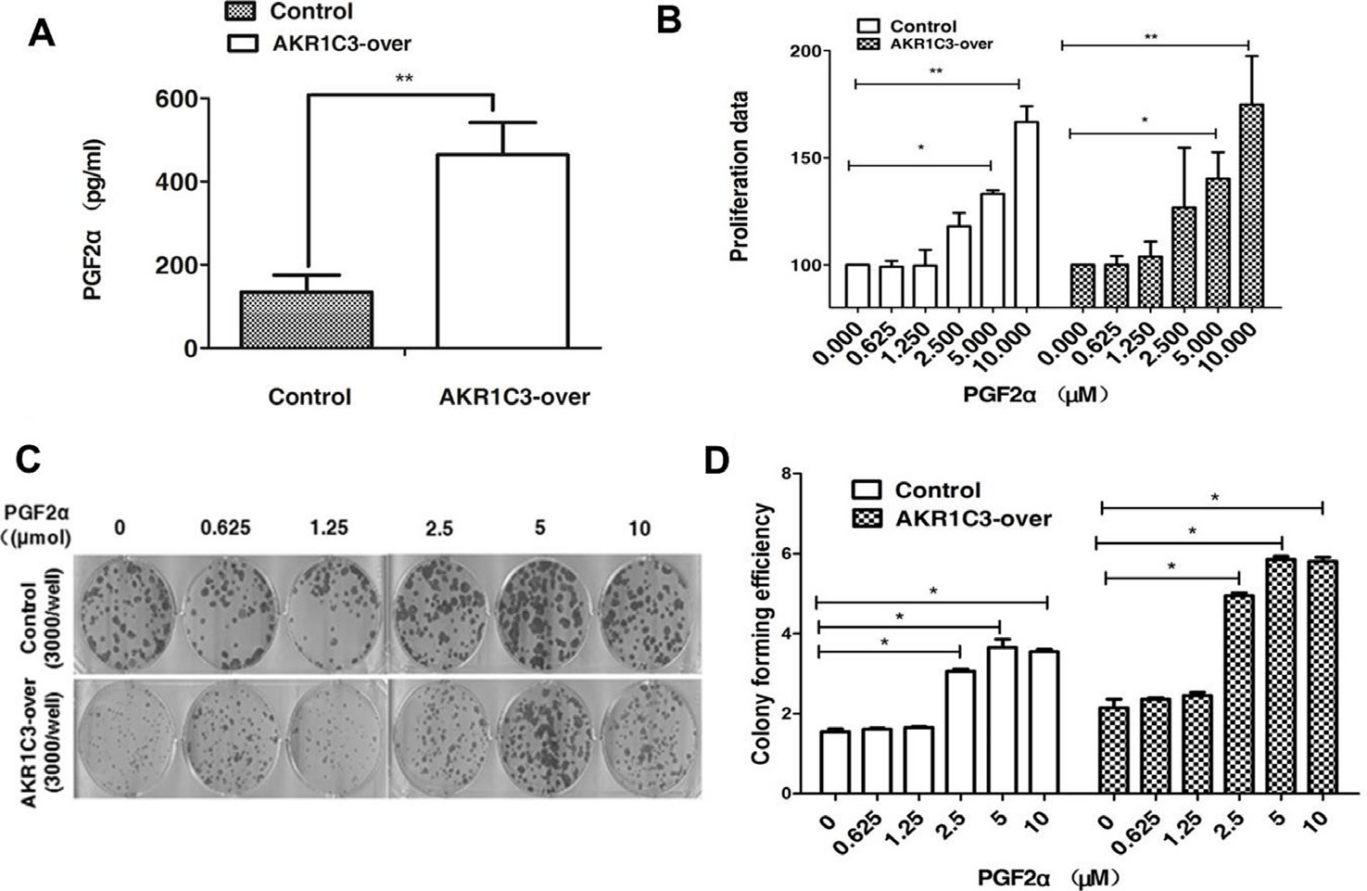
Overexpression of AKR1C3 in DU145 cells increases the amount of PGF2α (**A**) Overexpression of AKR1C3 in DU145 cells increases the amount of PGF2α. Cells were treated with 6 GY radiation and the concentration of PGF2α in supernatants was measured by ELISA for the indicated times (*n* = 2). *P < 0.05, **P <0.01. (**B**) The effects of PGF2α itself on cell proliferation: Control and AKR1C3-over cells were treated with the indicated concentration of PGF2α or DMSO for 6 days followed by cell proliferation assessment by CCK-8 assay. Data presented is the average of three experiments (SD), *P < 0.05, **P < 0.01. (**C**) The effect of PGF2α on cell radiationresistance was determined by clonogenic assay. The number of colonies formed in the wells increased as the PGF2α concentration increased. (**D**) Colony forming efficiency were calculated and results are presented as means SD of two experiments performed in duplicate.

**Figure 5:**
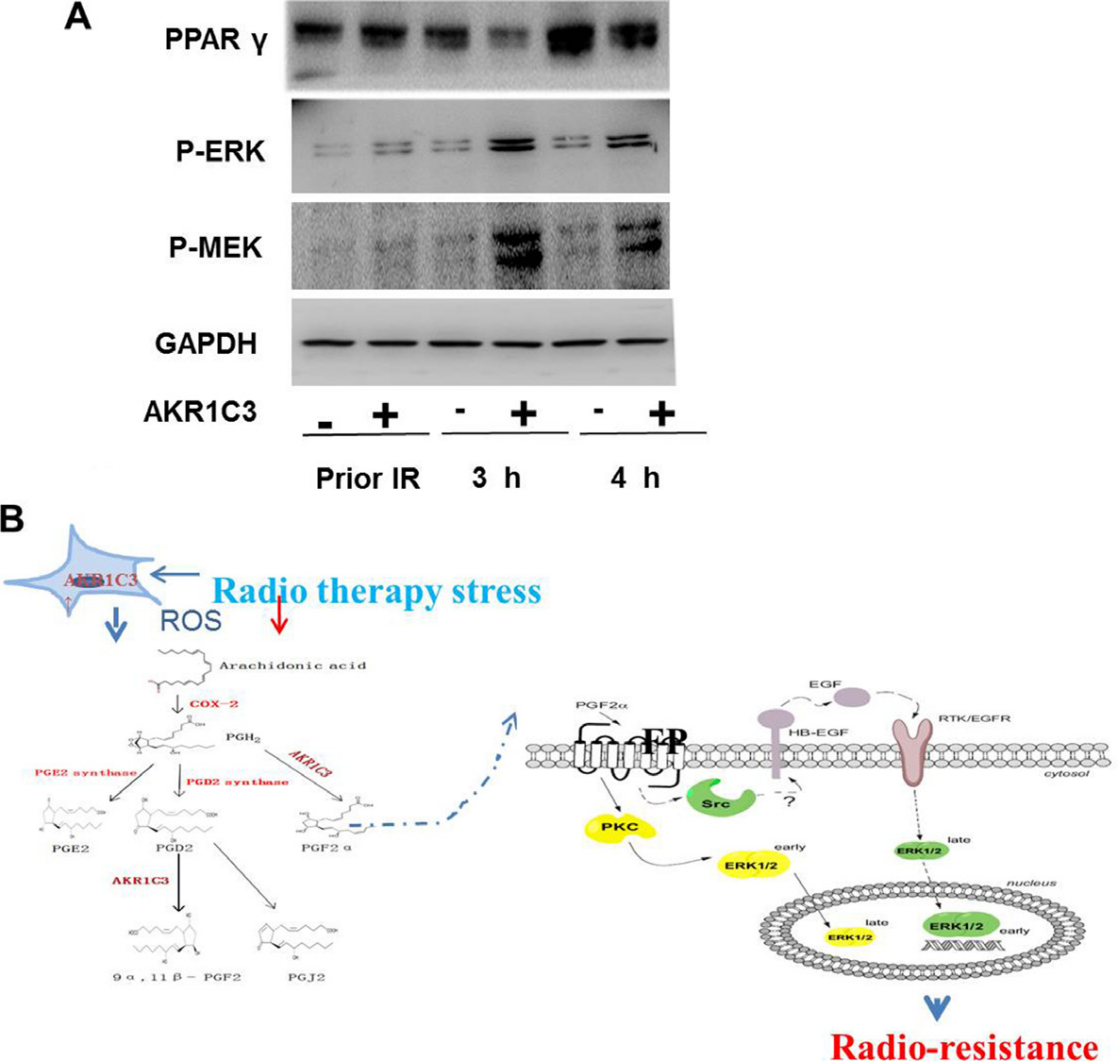
The accumulation of PGF2α activated the MAPK pathway and inhibited the expression of PPARγ (**A**) Western blotting results show that PGF2α augmenting the MAPK signaling pathway and inhibit the activation of PPARγ. (**B**) A proposed mechanism of AKR1C3-mediated radioresistance in DU145-over cells.

We evaluated the amount of PGF2α of control cells and AKR1C3-over cells after radiation through ELISA. Concentration of PGF2α were markedly higher in supernatants of AKR1C3-over cells compared with control cells at 3 hours after radiation (Figure 4A), so we speculated whether PGF2α played an important role in AKR1C3-over' s resistance to radiation. First, cell proliferation of both Control cells and AKR1C3-over cells was evaluated in the complete medium with PGF2α over a period of 6 days. PGF2α can exhibite significantly elevated cell proliferation at the appropriate concentrations both on Control and AKR1C3-over cells (Figure 4B). PGF2α has a proliferation effects on prostate cancer cells, to investiagte whether it has an effect on radiation resistance, we performed the clonogenic assay. Both Control and AKR1C3-over cells were pretreated with a series concentration of PGF2α after 6 gy radiation. Results showed that both Control and AKR1C3-over cells formed more colonies when cells were pretreated with PGF2α and the number of colonies increased when the concentration of PGF2α increased (Figure 4C and 4D). Results confirmed that PGF2α not only could promote prostate cancer cell's proliferation but also enhance prostate cancer cells resistance to radition. To explore the downstream signaling pathway by which prostaglandin F2α augmented, we dedected the involvement of MAPK signaling pathway and the PPARγ. Because the PGF2α receptor can activiate the MAPK pathway while the activiation of the MAPK pathway can inhibit the PPARγ pathway. PPARγ pathway are tightly associated with cell viability and proliferation.We examined the PPARγ and phosphorylation status of p-MEK, p-ERK by Western blotting. As shown in Figure 5A, AKR1C3-over after radiation for 3 hours and 4 hours resulted in an increase of p-MEK and p- ERK and a suppression of the PPARγ compared with Control.

## DISCUSSION

AKR1C3 has closed relationship with many tumours [14, 15, 23–25],such as breast cancer, colon cancer and choriocarcinoma [23, 25, 26], yet the role of AKR1C3 in prostate cancer cells radiation resistance has not yet been clarified. It was the first time that we found that AKR1C3 could result in PCa cancer cells 'radiation resistance. AKR1C3 plays an important role in androgen biosynthesis in prostate cancer cells to promote the progression of PCa and its resistance to hormone therapy [18, 27, 28]. Previous studies mainly focused on the aberrant expression of AKR1C3 and its biological activity in castrate resistant prostate cancer (CRPC) [29], whereas the potential effects AKR1C3 gene exerts on the radioresiatance of PCa was unclear. In the present study, for the first time, we showed that AKR1C3 was a radioresiatance related gene in PCa and it functioned through elimination of ROS and accumulation of PGF2α. Furthermore, these data confirmed the results of our previous work in esophageal cancer [14], showing AKR1C3 could also cause radioresistance in PCa, besides esophageal cancer [14] and NSCLC [15].

Radiotherapy remains the most effective nonsurgical treatment for most solid tumors and half of all cancer patients receive radiation as a part of their treatment. However, radioresistance remains a poorly elucidated phenomenon which might be congenitally intrinsic or induced by irradiation itself with the acquired resistance being genetically inherited. Identification of genes involved in the etiology of radioresistance should provide biomarkers for diagnosis of non-susceptible patients to radiotherapy.

AKR1C3 has gained extensive attention because of its potential involvement in affecting chemotherapeutic efficacy and inhibiting differentiation of tumor cells [23, 25]. Based on the findings from this study, we proposed that cellular redox-directed intervention via AKR1C3 might, to some extent, overcome radio- -resistance promoting the differentiation of tumor cells, providing a new strategy for the treatment of malignancy.

PGF2α can exerts its autocrine or paracrine functions through a G-protein-coupled receptor (FP)-mediated interaction [30, 31]. It has been reported that elevated expression of FP in human endometrial adenocarcinomas enhances the proliferation of endometrial epithelial cells [30, 31]. So, more experiments need to be done, such as the dedection of the expression in Control cells and DU145-over cells. The mechanism of the activation of intracellular signaling pathway by PGF2α-regulated is not yet clear. It has repoted that PGF2 could activate MEK/extracellular signal-regulated kinase (ERK) signaling pathways to regulate cell proliferation [32, 33] and angiogenic factor expression [34]. Other reports found that PGF2α -activated signaling pathway is mediated through ERKdependent but Akt-independent pathway [35]. We found that the PGF2α-activated MAPK activation resulted from the accumulation of PGF2 in DU145-over cells. MAPK pathway had been reported to contribute to the invasive potential of cancer cells [36] Also the activation of ERK/MEPK has been been suggested to be a key [37] There were several limitions need to be addressed. First, AKR1C3 is a hormones and prostaglandins enzyme which efficiently converts PGH2 to PGF2α and PGD2 to 9α, 11β-PGF2. In this article, we had just explored theAKR1C3 and PGF2α. If there were other prostaglandins participating in prostate cancer ‘radiation resistance warrants further studying. Second, to inhibit the activity of AKR1C3, we used indomethacin, a widely used inhibitor for AKR1C3 in previous studies [20–22, 38], however, we could not exclude the possibility that the effects of AKR1C3 inhibition on cell growth was caused by indomethacin itself. Therefore, further investigations are warranted to clarify this issue.

In our experiment we used DU145 cells to generate a cell line that stably overexpresses AKR1C3 by means of retroviral infection. The DU145 cell line was infected with either AKR1C3 overexpressing retroviral construct (AKR1C3-over) or with anempty vector (Control). The levels of AKR1C3 overexpression in AKR1C3-over cells was confirmed by Western blot analysis (Figure 1A). Cell clone experiments confirmed AKR1C3 overexpressing DU145 cells are resistant to radiation (Figure 1B–1C), And Suppression of AKR1C3 via its chemical inhibitor indocin restored the sensitivity of the acquired tumor cells (Figure 2A–2D). AKR1C3 can alleviate oxidative stress (Figure 3A–3D). Also overexpression of AKR1C3 can resulted in the accumulation of prostaglandin F2α (Figure 4A). Then, the effect of PGF2α on radiation resistance was evaluated by colony formation assay. The number of colonies formed in the wells increased as the PGF2α concentration increased (Figure 4C–4D). This resistance to radiation suggests that AKR1C3 overexpression in DU145 may protect tumors from the antiproliferative effects mediated by this PG. At last, we measured its key downstream pathway. Western blotting results show that PGF2α augmenting the MAPK signaling pathway and inhibit the activitiation of PPARγ (Figure 5A).

## MATERIALS AND METHODS

### Cell culture

The DU145 prostate cancer cell line was provided by the Urinary Surgery Department of the First Affiliated Hospital of Peking University. Cells were cultured in RPMI 1640 medium (M&C Gene Technology, Beijing, China) supplemented with 10% fetal bovine serum (FBS, Gibco, Auckland, New Zealand). Cells were maintained in a humidified incubator with 5% CO2 at 37°C.

### Overexpression of AKR1C3

Construction of AKR1C3-over (BamHI-AKR1C3-BamHI) and control (BglII-CMV-BamHI) cassettes were performed by PCR. The BglII-CMV-BamHI sequence were:

3.1-CMV-for(bglII):GAAGATCTgttgacattgattattgac; 3.1-CMV-rev(BamHI):CGGGATCCctagccagcttgggtctc; and the BamHI-AKR1C3-BamHI sequence were: BamHI-AKR1C3-for:CGGGATCCgccaccatggattc; CMV-AKR1C3-rev(BamHI):CGGGATCCttaatattcatctgaata. Cell culture, lentivector package, and stable transduction as were performed as previously described [19].

### Ionizing radiation treatment

Irradiations were performed with a medical linear accelerator (Varian Clinic 23EX, Varian Medical Systems, USA) using 6 MV photons with an absorption dose rate of 4 Gy/min. All tumor cell irradiations were performed at the Department of Radiation Oncology of Peking University First Hospital.

### Prostaglandins assessment

Prostaglandins concentration was assessed by ELISA (Cayman Chemical, Ann Arbor, MI, USA). Cells were seeded in six-well plates and allowed to reach 90% confluence. Cells were treated with 6MV-X-ray in growth medium without supplements for the indicated times. Prostaglandins in each condition was determined in supernatants by ELISA following manufacturer directions. Each condition was evaluated for prostaglandins by averaging a minimum of two OD measurements.

### Cell proliferation assessment by CCK-8 assay

Cell proliferation was determined using CCK-8 assay according to the protocol provided (CK-04; Dojindo). To evaluate the effect of prostaglandins on cell proliferation,cells were plated in 96-well plates in growth media in medium containing 0 μM, 0.625 μM, 1.25 μM, 2.5 μM, 5 μM, 10 μM prostaglandins (PGFα) for 7 days. Then each condition was evaluated by averaging a minimum of three OD measurements.

### Colony formation assay

Cells at the exponential stage of growth were then exposed to different dosages of radiation (0, 2, 4, 6, 8 Gy) and the irradiated cells were seeded in six-well plates. After incubation for 7–14 days, the surviving cells were fixed with methanol and stained with crystal violet, and colonies containing more than 50 cells were counted. Each operation was repeated twice.

### Flow cytometry assay

Flow cytometry measurements of DFCH-DA were used to measure cellular ROS levels. AKR1C3-over and Control cells were seeded in six well cultured to 60% confluence. The cells were then treated for 15 min with t-BHP and then 10 mM DCFH-DA(D6883/50 mg, Sigma) and hoechst probe for 15 mins immediately after loading, the cells were washed with ice-cold PBS for three times and visualized by fluorescence microscopy (IX81, Olympus, Japan). The mean fluorescence intensity (MFI) was calculated after correction for autofluorescence and the foldchange was calculated. Each flow cytometry assay was repeated twice. The initial parameters were analyzed in the cellular imaging & analysis system (Columbus 2.4, PerkinElmer, Waltham, Massachusetts, USA).

### Western blot analysis

Protein expression and phosphorylation in DU145 cells treated with either 0 GY (control) or 6 GY radiation were evaluated using Western blotting. Briefly, DU145 cells were seeded in 6-well plates and cultured to 80% confluence. The cells were then treated with 0 GY (control) or 6 GY radiation and incubated for 48 h, 72 h and 96 h. Then cell protein extracts were prepared. For all experiments 30 ug protein was used and the primary antibodies were diluted as follows: Anti-AKR1C3 1:1000 (A6229–200U;Sigma);anti-humanGAPDH1:3000;anti-phospho-ERK1/2-T202/Y204:1:1000(AP0472;Abclonal);anti-phospho-MEK1/2 (Ser217/221):1:1000(#9121;CST);anti-PPARγ:1:1000 (A0270; Abclonal), Protein extraction and western blot procedures were carried out as previously described [14]. Each Western blotting was repeated at least twice.

### Statistical analysis

All statistical analyses were performed using SPSS 19.0 (SPSS, Chicago, IL, USA) and GraphPad5 software. The Student's *t*-Test was used to determine the statistical difference between means of two groups and data. *P* < 0.05 were considered as significant.

## CONCLUSION

In our study, we found that overexpression of AKR1C3 in PCa could result in radioresistance through elimination of ROS and accumulation of PGF2α, which could not only promote prostate cancer cells' proliferation but also enhance prostate cancer cells' resiatance to radition. The accumulation of PGF2α activated the MAPK pathway and inhibited the expression of PPARγ (Figure 5B).
