# Correction: Overexpression of AKR1C3 significantly enhances human prostate cancer cells resistance to radiation

**DOI:** 10.18632/oncotarget.27542

**Published:** 2020-04-28

**Authors:** Shao-Qian Sun, Xiaobin Gu, Xian-Shu Gao, Yi Li, Hongliang Yu, Wei Xiong, Hao Yu, Wen Wang, Yingbo Li, Yingqi Teng, Demin Zhou

**Affiliations:** ^1^ Department of Radiation Oncology, Peking University First Hospital, Peking University, Beijing, China; ^2^ State Key Laboratory of Natural and Biomimetic Drugs, School of Pharmaceutical Sciences, Peking University, Beijing, China; ^3^ Department of Radiation Oncology, Jiangsu Cancer Hospital Affiliated with Nanjing Medical University, Nanjing, China; ^4^ Tangshan People’s Hospital, Hebei, China; ^5^ Beijing Reciproca Pharmaceutical Co. Ltd., Beijing, China

**This article has been corrected:** In Figure 2C, in the column labeled ‘4GY’, the picture of Indocin- is mistakenly identical to the picture for Indocin+. The corrected Figure 2C, obtained using original data, is shown below. The authors declare that these corrections do not change the results or conclusions of this paper.

Original article: Oncotarget. 2016; 7:48050–48058. 48050-48058. https://doi.org/10.18632/oncotarget.10347

**Figure 2:**
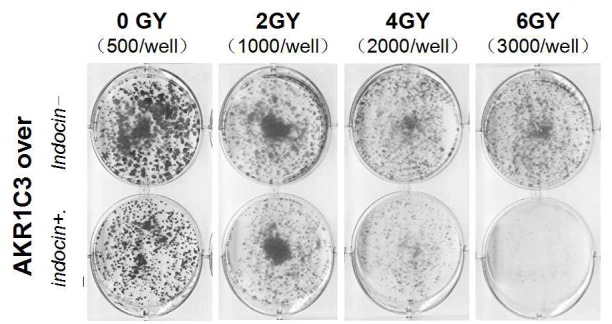
Indomethacin, an inhibitor of AKR1C3 activity, overcomes radiation resistance.

(**A**) AKR1C3-over cells and control cells were treated with or without 20 mmol/ indomethacin for 2 days, and western blotting was performed; (**B**, **C**) AKR1C3-over cells and Control cells were treated with or without 20 mmol/ indomethacin for 2 days, and clonogenic assay was performed; (**D**) Colony forming efficiency were calculated and results are presented as means SD of two experiments performed in duplicate.

